# Modelling upper respiratory tract diseases: getting grips on host-microbe interactions in chronic rhinosinusitis using in vitro technologies

**DOI:** 10.1186/s40168-018-0462-z

**Published:** 2018-04-24

**Authors:** Charlotte De Rudder, Marta Calatayud Arroyo, Sarah Lebeer, Tom Van de Wiele

**Affiliations:** 1 Center for Microbial Ecology and Technology, Faculty of Bioscience Engineering, Ghent University, Coupure Links 653, B-9000 Ghent, Belgium; 2 Research Group of Environmental Ecology and Applied Microbiology, Department of Bioscience Engineering, University of Antwerp, Groenenborgerlaan 171, 2020 Antwerp, Belgium

## Abstract

Chronic rhinosinusitis (CRS) is a chronic inflammation of the mucosa of the nose and paranasal sinuses affecting approximately 11% of the adult population in Europe. Inadequate immune responses, as well as a dysbiosis of the sinonasal microbiota, have been put forward as aetiological factors of the disease. However, despite the prevalence of this disease, there is no consensus on the aetiology and mechanisms of pathogenesis of CRS. Further research requires in vitro models mimicking the healthy and diseased host environment along with the sinonasal microbiota. This review aims to provide an overview of CRS model systems and proposes in vitro modelling strategies to conduct mechanistic research in an ecological framework on the sinonasal microbiota and its interactions with the host in health and CRS.

## Background

The upper respiratory tract is one of the primary body sites of contact with the outside environment and its physical, chemical and infectious agents. It is therefore unsurprising that upper respiratory tract infections are a major health concern. Chronic rhinosinusitis (CRS) is a disease that affects 10.9% of the adult population in Europe [1]. It is defined as chronic inflammation (*>* 12 weeks) of the nasal and paranasal sinus mucosa, characterised by two or more symptoms, one of which should be either nasal obstruction, congestion or nasal discharge and is further identified by facial pain, pressure and/or reduction or loss of smell [2]. Supportive objective evidence includes rhinoscopic or endoscopic findings of polyps and/or purulent discharge and/or oedema or mucosal obstruction in the middle meatus and/or ethmoid region. Next to this, a CT scan can demonstrate mucosal changes within the paranasal sinuses [3]. CRS patients are typically classified in two major clinical subgroups or phenotypes based on the presence or absence of nasal polyps (NP) (respectively CRSwNP and CRSsNP) [2, 3], although endotypes and clusters can be distinguished within these subgroups, based on inflammatory profiles [3, 4]. Despite the high prevalence of CRS, there is no consensus about the aetiology of the disease [5]. In recent years, however, understanding of the importance of the sinonasal microbiome in this disease is emerging [6–10]. The view that a respiratory microbiota in equilibrium contributes to host health by immune priming and provision of colonisation resistance, similar to the gut microbiome, gains interest [11–14]. Analogously, a disrupted airway microbiota could decrease resistance to pathogen infection and overgrowth and result in inflammatory responses [10, 15, 16]. Culture-independent techniques, such as next-generation sequencing, have enabled in-depth study of the differences in community structure in healthy and CRS upper respiratory tracts [7, 17–25].

In addition to in vivo human research, mouse, rabbit and sheep models to further study CRS have been developed, despite the ambiguous aetiology of the disease (as reviewed in Shin [26]). Current animal models are either based on infection with specific bacterial or fungal pathogens [11, 27] or on immunostimulation causing allergic rhinosinusitis [28, 29]. In vivo animal models of CRS can be used to study more complex immune responses, pathogen invasion in a natural microbiota context, and histologic and systemic effects [11, 29, 30] than in vitro models. Differences in sinus morphology, disease development, microbiome, pathogens and immune response between humans and test animals should be taken into account in the interpretation of results [26]. Although in vivo observations and intervention studies supply valuable information, there is a need for in vitro models according to the 3R principle (refinement, reduction, replacement). Adequate in vitro models mimicking the in vivo environment allow initial testing of numerous conditions such as effects of pathogen or allergen exposure on the host, influence of physical changes in the host microenvironment on both host and microbiota or exploration of the potency of probiotics or biologicals in disease prevention and/or control. Subsequently, the most promising settings can be selected for animal studies, hereby refining the in vivo research and reducing the required number of laboratory animals [31]. To study host-microbe interactions in health and disease, systems mimicking the host mucosal environment and its complex microbiota in a reproducible manner have recently been developed, i.e. in our research group, for the human gastro-intestinal tract [32–34] and the oral cavity [35]. In order to study airway diseases and mechanistically understand host-microbe interactions, the development of similar, representative models for the respiratory tract is essential.

In this review, a short overview of the main characteristics of the sinonasal CRS environment is given (the reader is referred to Hamilos [36], Hamilos [37], Scheckenbach and Wagenmann [38] and Stevens et al. [39] for reviews on the immune response in CRS and to Mahdavinia et al. [40] and Hoggard et al. [41] for the microbiota in CRS). Furthermore, we aim to review the current methods to study airway host-microbe interactions in vitro and propose in vitro modelling strategies to conduct mechanistic research in an ecological framework on the sinonasal microbiota and its interactions with the host in health and CRS.

## The sinonasal microenvironment

### Epithelial lining of the sinonasal cavities

The nose and paranasal sinuses (the maxillary sinuses, the ethmoid sinuses, the sphenoid sinuses and the frontal sinuses) form a specific niche within the human body. They consist of a unified system of skin and mucosal surfaces and form the primary site of contact with inhaled air in the respiratory tract. The nostrils and anterior nares are lined with keratinized stratified squamous epithelium and contain serous and sebaceous glands [42]. The surfaces of the nasal cavity and the paranasal sinuses on the other hand are carpeted with typical respiratory epithelium with pseudostratified ciliated columnar epithelium and are characterised by the presence of basal cells and mucin-secreting goblet cells [43]. The activity of the goblet cells results in the presence of a mucus layer, which is continuously removed by mucociliary clearance and subsequently collected in the nasal cavity.

### CRS-associated sinonasal microenvironment

The inflammatory state of the sinonasal epithelium of CRS patients renders their sinonasal cavities different from those in healthy individuals. Inflammatory tissue is often characterised by hypoxia, a low level of oxygen in tissues, typically occurring when available oxygen levels are between 0.5 and 3% [44], and altered interleukin (IL) secretion (e.g. IL-13), both factors inducing goblet cell hyperplasia [45, 46], mucus hypersecretion [46–48] and increased mucin expression [45–47]. These factors result in increased thickness of the mucus layer and mucostasis, a lack of mucus flow resulting in mucus accumulation. Furthermore, the diameters of the ostia, the openings connecting the sinus cavities with the nasal cavity, can be severely reduced or completely obstructed. Leung et al. [49] found uni- or bilateral osteomeatal complex obstruction in 64.4% of CRSsNP patients (*n* = 144) and in 75.0% in CRSwNP patients (*n* = 123), from a patient population requiring surgery for CRS. This causes a reduction in sinus aeration (hypoxia) and an additional accumulation of mucus and other sinus secretions. Hypoxia itself induces tissue remodelling (e.g. polyposis) and upregulation of inflammatory pathways [44, 50]. Hypoxia can decrease the expression of antimicrobial proteins [51], facilitate the growth of anaerobic bacteria [52] and eventually result in increased inflammatory tissue damage [53].

An improper epithelial barrier function, with increased paracellular permeability and decreased tight junction expression, has also been linked to the CRS disease phenotype [54–56]. Possibly due to the impaired barrier function of damaged epithelium, CRS patients have significantly increased nasal airway surface liquid (ASL) glucose concentrations compared to control patients [57]. In contrast, carbon sources, such as glucose, are actively depleted from ASL by healthy airway epithelium [58]. The increased glucose concentration in CRS ASL might facilitate bacterial growth by increased carbon source availability and reduced antimicrobial protein secretion via a bitter taste receptor (type 2 receptor)-mediated pathway [57]. Bitter taste receptors have recently been identified as players in innate sinonasal immunity against bacterial invasion and biofilm formation through nitric oxide production, and increased ciliary beating upon activation by bacterial quorum sensing molecules and their (dys)function could play a role in CRS recalcitrance [59–62].

**Fig. 1:**
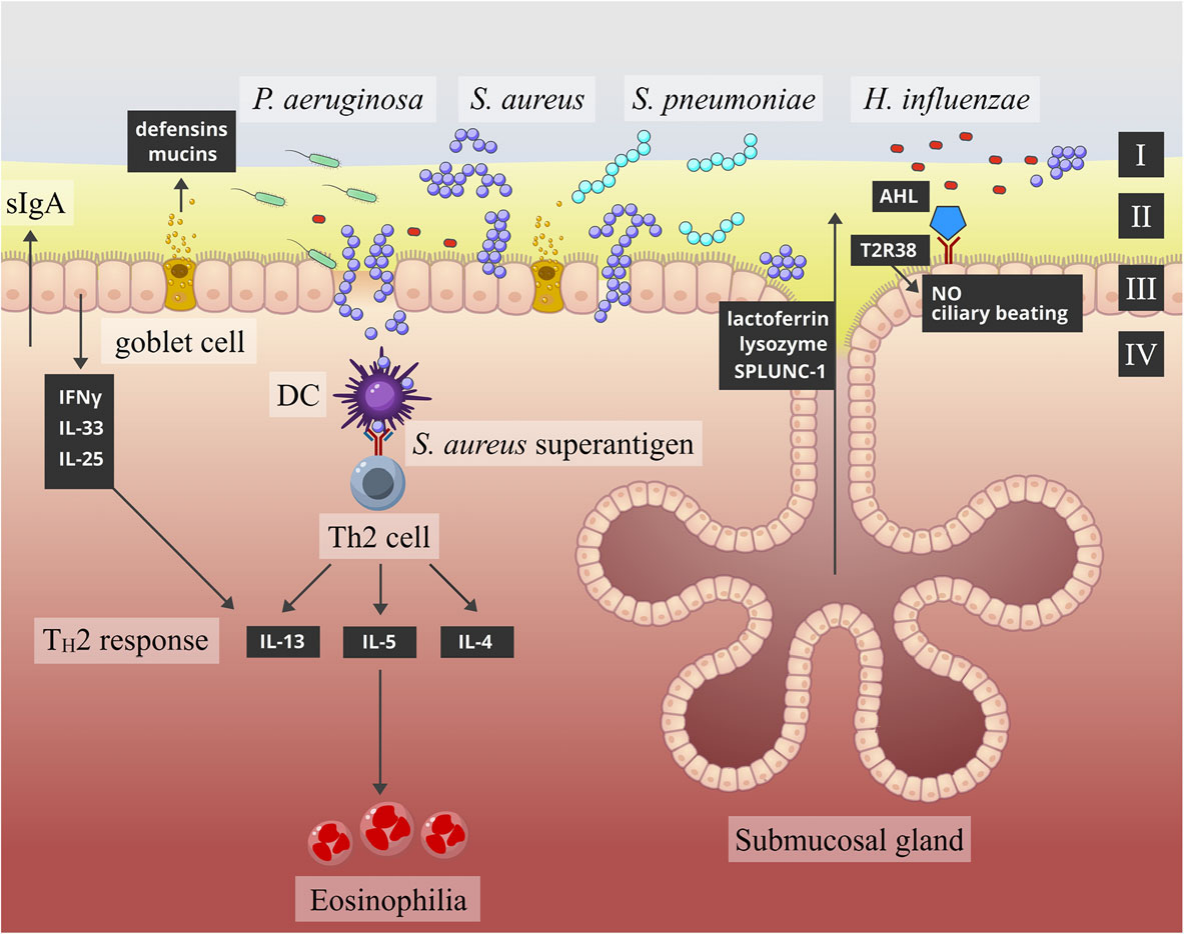
Schematic representation of CRS tissue. A proposed model for the changes of the sinonasal environment during CRS. Increased mucus production and/or goblet cell hyperplasia can result in a mucus layer with increased thickness (I). Changes in sinus microbiota can be observed, more specifically the abundances of CRS-associated pathogens *S. aureus*, *P. aeruginosa*, *S. pneumoniae* and *H. influenza*. Proposed changes involve decreased epithelial integrity (III: defective epithelial barrier function), with increased glucose concentrations in the airway surface liquid (II), decreased recognition of acyl-homoserine lactones by T2R38 receptors, altered cytokine production and decreased antimicrobial protein and immunoglobulin production that occur in CRS compared to healthy tissue. These changes potentially provide an environment that promotes pathogenic bacterial growth and invasion of the epithelial barrier (IV), resulting in chronic inflammation. *S. aureus* has the ability to produce a super-antigen, which evades conventional immune response and directly elicits eosinophilia and a T_*H*_2-skewed immune response (IL-5, IL-13, IL-4), whereas IFN-γ induces a T_*H*_1-skewed immune response (figure adapted from Mahdavinia et al. [40])

The production of antimicrobial proteins (AMP), such as lysozyme, lactoferrin, PLUNC (palate, lung, and nasal epithelial clone) proteins and defensins, and the secretion of immunoglobulin A (IgA) are protective mechanisms in the upper respiratory tract (URT) [63]. Altered profiles of these compounds have been associated with CRS pathogenesis. Decreased short PLUNC-1 (SPLUNC-1) has been observed in CRSwNP [64] and CRSwNP polyp tissue, compared to control tissues [65]. Decreased SPLUNC-1 also appeared to be associated with positive *Pseudomonas aeruginosa* bacterial colonisation and poor clinical outcomes [64, 66]. It has been demonstrated in nasal polyp cultures at air-liquid interface (ALI) that IL-13 has a downregulating effect on SPLUNC-1 expression [67]. Next to AMP, the epithelium has the ability to produce a broad array of cytokines and chemokines to modulate immune responses towards both commensal and invading microorganisms. Various studies have yet shown altered cytokine profiles in cell cultures from CRS patients compared to non-CRS controls [54, 68, 69]. Distinctive cytokine profiles are observed between CRSwNP and CRSsNP [2] and the different endotypes therein [3, 4]. CRSsNP appears to display a T_*H*_1-skewed cytokine profile, while CRSwNP is more commonly characterised by a T_*H*_2 response and eosinophilic NP in Caucasian patients [70, 71]. In contrast, an interferon-*γ* (IFN-*γ*)- and/or IL-17-biased profile with neutrophilic NP appears more typical in Asian CRSwNP patient populations [72]. An overview of the most outstanding parameters of the CRS mucosal environment is presented in Fig. 1.

## Bacterial microbiome of the sinonasal cavities

### Composition of the sinonasal bacterial communities

In recent years, interest in the sinonasal cavity microbiome has increased, and the use of molecular methods to study microbial communities has enabled characterisation of the sinonasal microbiota, bypassing cultivation bias [7, 17–25]. They show that the sinonasal microbiota is very diverse and highly variable between individuals [7, 17–25] and indicate a high temporal variability within individuals [17].

The microbiome of the sinonasal cavities appears to be characterised by the high relative abundance of three phyla: Firmicutes, Actinobacteria and Proteobacteria [17–19, 25]. *Corynebacterium* and *Propionibacterium* species have been found at high abundances in most individuals [17, 18, 24, 25, 73]. The species richness in the nasopharynx of healthy westernised adults varied greatly between subjects and time points and has been estimated (Chao1 index) to be between 6 and 110 (average = 50, *n* = 97) [17]. Yan et al. [25] reported estimated species richness (Chao1 index) in the anterior naris, middle meatus and sphenoethmoidal recess between 200 and 400 (*n* = 179). Healthy sinonasal cavities are relatively nutrient-poor niches in the human body for microbial colonisation [74]. As a result, the bacterial density in the sinonasal cavities is estimated to be at least 10 times lower in comparison to the oral cavity (based on 16S copy numbers in sample) [75].

### Dysbiosis in CRS

Dysbiosis of the bacterial community composition, a disruption of the equilibrium and mutualistic relationships between commensal microorganisms mutually and with the host epithelium, has been implied in the aetiology, pathogenesis and recalcitrance of CRS [9–11, 13, 41]. Mackenzie et al. [10] suggested that the bacterial community associated with CRS is dysbiotic and that the ecological networks in these communities are fragmented. They proposed *Burkholderia* and *Propionibacterium* as keystone genera in healthy ecological networks [10]. It remains however unclear whether an imbalance in the local microbiota and/or colonisation by pathogenic species elicits an immune response and results in a chronic inflammatory condition or whether an initial inflammation of the epithelium and/or sinus obstruction provides a platform for sustained bacterial dysbiosis, resulting in a CRS phenotype [6, 37, 63]. In addition, the interplay between the (inadequate) host immune response and the microbiota (in a state of dysbiosis) might also be explanatory for the CRS phenotype [76]. Apart from the causality of dysbiosis for CRS, the sinonasal microbiome could play a role in the self-perpetuation of the disease and be decisive in the therapeutic outcome. Several studies have suggested that the diseased CRS sinonasal environment is characterised by a higher bacterial load and/or a lower diversity. However, contradictory results have been observed. Several research groups [11, 20, 72, 77] detected no difference in bacterial load between CRS and non-CRS patients, although a reduced diversity was observed in CRS patients. Contrarily, a higher bacterial load [7, 73] and increased diversity [6] were observed in other studies comparing (refractory) CRS patients to non-CRS controls. Aurora et al. [6] analysed nasal lavage fluid of CRSwNP, CRSsNP and control patients, which, contradictorily to the observations of Aurora et al. [6], revealed a lower species diversity and an increased bacterial burden in CRS patients compared to controls. By comparing the bacterial load in CRS and non-CRS patients, it has been proposed that the bacterial load in the sinonasal cavity is defined, but that the community composition is rather influenced by conditions such as illness and antibiotic use [11, 72, 78]. The lack of consensus on a specific bacterial profile associated with CRS could be a result of the diversity of DNA collection method and the sampling place within the sinonasal environment. Furthermore, a history of antibiotic use in CRS, the phenotype and severity of the disease, and disease subgroups can be confounding factors. Typically, only small patient and control cohorts (e.g. respectively 9 and 6, [20]) can be used for sinus microbiota sampling due to the requirements to stay off antibiotics for 4 weeks prior to surgery and the difficulty of sampling via endoscopic sinus surgery [20]. The most important confounding factors are antibiotic and corticosteroid use in case of CRS, as these are known to severely impact microbiota diversity [22, 79]. Biswas et al. [20] selected CRS patients that were off antibiotics for 4 weeks prior to surgery. The impact of this selection criterion is however unclear, as Feazel et al. [22] reported a significant reduction in species richness and evenness as a result of antibiotic use 12 weeks prior to sampling. The CRS patients sampled by Abreu et al. [11] all had a history of antibiotic use.

### Biofilm formation in CRS

Next to their community composition, the physiological state in which bacteria are present on the mucosal surface has importance in the CRS context. Biofilm formation is a commonly observed survival strategy among microorganisms in which a microbial community is enclosed in an exopolymer matrix of their own making and attached to a surface. Biofilms can have a complex structural organisation, which enables intense interactions between the composing organisms. They provide protection from physicochemical environmental stressors such as shear stress and harmful compounds. Especially relevant in a healthcare context is the enhanced antibiotic resistance observed in biofilms, which can be a factor of 1000 higher than the same species in planktonic form [80, 81]. Chronic disease, extreme resistance to antibiotic treatment and repeated acute exacerbations are characteristic of biofilm-mediated diseases and are also observed in CRS patients [70, 82].

Biofilm formation in CRS can be confirmed by electron or laser microscopy on tissue biopsies [73, 83] and has been associated with more severe disease and worse treatment outcome after endoscopic sinus surgery in comparison with non-biofilm CRS [84, 85].

## Modelling the URT microenvironment and microbiota

**Fig. 2:**
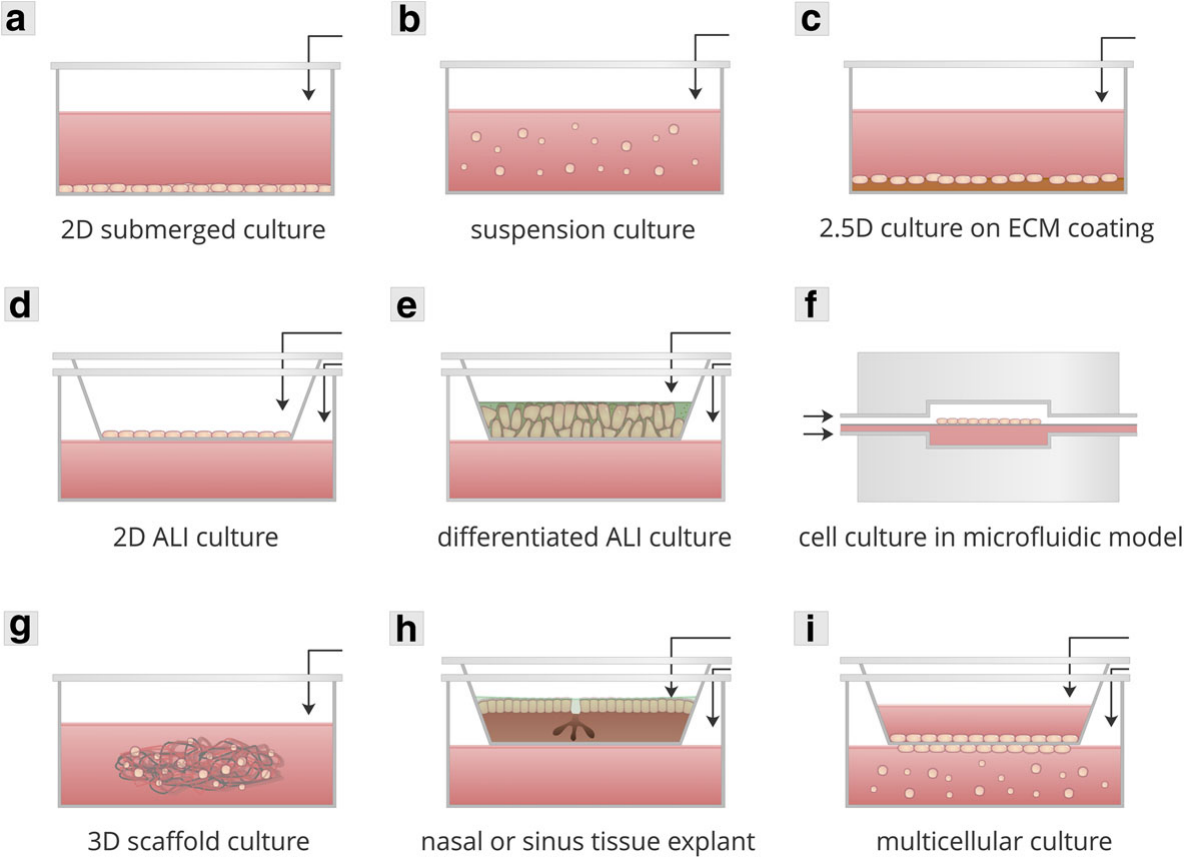
Schematic overview of the categories of cell culture used to model airway tissue and host-microbe interaction in the airways. Black arrows indicate where addition of bacteria, bacterial cell wall compounds and/or other antigens is possible to mimic host-microbe interaction. **a** Two-dimensional (2D) submerged culture, also known as conventional monolayers. Cells are typically cultured directly on a substrate such as plastic. **b** Suspension culture of non-adherent cells, e.g. undifferentiated immune cells. **c** Two and one-half-dimensional (2.5D) culture. Cells are cultured on top of a layer of ECM. **d** 2D culture at air-liquid interface (ALI). During ALI conditions, cells cultured on porous (membrane) inserts are deprived of medium in the apical compartment, exposing them to the atmosphere and requiring them to take up nutrients from the basolateral compartment. **e** Differentiated ALI culture. Histologically realistic epithelial tissues can be constructed in stages, with initial assembly of airway epithelial cells into an epithelial cell layer (2D monolayer) on a submerged culture insert, followed by exposure of these cells to an air-liquid interface during a period of 2–4 weeks to induce the formation of polarised, pseudostratified airway epithelium with basal cells, ciliated cells and mucin-secreting goblet cells. **f** Cell culture in microfluidic model. ALI culture in a microfluidic model is an approach that mimics the in vivo environment more closely than static cell culture models. **g** 3D scaffold culture. Cells are cultured in and/or on biomimetic scaffolds, submerged or at ALI. **h** Nasal or sinus tissue explants. Excised nose or sinus epithelium from patients or healthy controls is cultured on a tissue culture insert that is either submerged in medium or maintained at an air-liquid interface. **i** Multicellular models. The combination of different cell types within a model is a strategy to more accurately represent the host microenvironment and the interactions therein. Airway epithelial cells can be combined with endothelial or immune cells to mimic host-microbe interaction on different levels

Representative in vitro models of the upper respiratory tract and its microbiota can provide an elegant platform to conduct mechanistic research on chronic rhinosinusitis. A proper in vitro model allows the exploration of a number of fundamental hypotheses in a simplified and controlled environment and selection of the conditions subsequently to be tested in vivo. Due to the ambiguous aetiology of CRS, an optimal model should comprise, in close interaction, host components as well as microbial components. An overview of these techniques is presented in Fig. 2.

### Requirement host interface

A first constituent of the host in vitro microenvironment is the respiratory epithelium lining the deeper sinonasal cavity. It forms the primary site of interaction with the external environment and its microbiota. Incoming microbes have to cope with the innate (and adaptive) immune defences during attachment and growth. Therefore, in vitro models should include a differentiated epithelial structure with ciliated and non-ciliated, goblet and basal cells, with the following functionalities: mucus production, ciliary movement, barrier function and cytokine, chemokine and AMP secretion. Furthermore, apical-basolateral differentiation is needed for bacterial attachment. Currently, the only in vitro models expressing these traits are differentiated ALI epithelial cell cultures and organotypic explants [86].

Another point of attention is the altered physical host conditions in a CRS environment. Mechanistic research to address the question whether inflammation versus infection causes CRS requires a model in which a diseased host environment can be induced to observe the effects of inflammation or defective immune defence to the resident microbiota. A functional immune component is a second requirement of a sinonasal host-microbe interaction (HMI) model. This condition could be met by the incorporation of immune cells [87]. Ideally, the T_*H*_2- or T_*H*_1/T_*H*_17-skewed immune responses in the CRS sub- and endotypes, as previously described [3, 4], should be comprised in the model.

### Requirement microbial interface

Research on hypotheses regarding the role of microorganisms in CRS aetiology and pathogenesis on the other hand requires a healthy host component reacting to an infecting pathogen or microbiota in dysbiosis. The microbial compartment of a CRS model should consist of a diverse, complex community, as observed in vivo. To sustain a representative in vitro sinonasal microbiome, a host compartment providing the correct microenvironment as well as a representative healthy or CRS inoculum is needed. As elaborated above, it is still not clear whether a CRS microbiota composition, distinctive from a healthy sinonasal microbiota, exists; therefore, it might be of more interest to investigate the functional differences between healthy and CRS communities.

In vitro CRS models are still at an early developmental phase. Existing models are limited to either host-only models, represented by ex vivo tissues, primary cell cultures and immortal cell lines, or microbe-only models. Microbial models for CRS lack the diverse community composition seen in the sinonasal environment but are rather focused on limited species competition or interaction assays (e.g. between *Staphylococcus aureus*, *Streptococcus pneumoniae* and *Haemophilus influenzae* [88] and between *S. pneumoniae* and *H. influenzae* [89]). The same restriction applies for in vitro modelling of host-microbe interactions in CRS as existing models are co-culture systems of host cells with one or two microbial species (e.g. airway epithelial cells with *S. aureus*) [68, 90]. These models allow investigation of specific inflammatory mechanisms, competition between microbial species and particular host-microbe exchanges. However, they suffer from a reduced complexity, unable to mimic the role of the commensal microbiota in this multifactorial disease. An overview of current in vitro models for CRS is given.

### Mimicking the host microenvironment

Interest in upper respiratory tract diseases has resulted in the development of multiple in vitro models to study the response of airway epithelium to allergen and antigen exposure. Models that can be used to mimic upper airway epithelium are two-dimensional monolayers (2D), differentiated pseudostratified cell layers either submerged or at air-liquid interface (ALI) (between 2D and 3D) and 3D cell culture models (co-cultures, explants and 3D scaffolds). Microporous polymer membranes and conventional plates are commonly used and can be adjusted with coatings (2.5D culture), most often collagen, to mimic the natural extracellular matrix [91]. Alternatively, biomimetic (electrospun) structures [87, 92] or decellularized tissues [93] can be used as substrates for cell culture. The choice of substrate is important as it can influence cell attachment, polarity, differentiation and barrier formation [91].

#### Cell types

Immortal cell lines commonly used to mimic the URT are 16HBE (human bronchial epithelial cells) and Calu-3 and A549 (cells from human lung carcinoma) [94–96]. Immortal cell lines have the advantages of easy acquisition, source reliability and low cost. The ability to repeat experiments and yield reproducible results is another benefit. Disadvantages of immortal cell lines are the altered geno- and phenotype compared to in vivo tissue and the inability to obtain a desired disease phenotype (e.g. CRS). Furthermore, tissue-specific material is not always available or adequate for use in particular experiments. Primary patient material, such as cells from nasal brushings and epithelial cells isolated from sinus tissue or nasal polyps, has the advantage of retaining an in vivo-like phenotype and can be both disease- and tissue-specific [54, 62, 68]. Disease-specific materials, such as nasal polyp-derived cells, model the impaired sinonasal epithelium more accurately in terms of paracellular permeability and ciliary movement [54]. Disadvantages of primary patient material are the source variability, the ethical issues with sampling, the high cost, the low reproducibility due to biological variation and the specialised laboratory personnel required. However, there has been an increase in the commercial availability of primary cells and disease-specific primary cells (e.g. American Type Culture Collection^®^), partly overcoming these drawbacks.

#### Conventional monolayers or suspension cultures

2D monolayers in conventional plastic plates and membrane inserts are the most commonly used models for airway epithelial cell cultures. They can be optimised with coatings (e.g. collagen), sometimes referred to as 2.5D cell culture [91]. They are easy in use and are relatively low in labour intensity, requirement for specialised laboratory personnel, time demand and cost. Airway epithelial cell monolayers are obtained by seeding cells on a substrate or scaffold and culturing them until fully confluent. 2D monolayers of, for example, but not limited to, primary human nasal epithelial cells can be repeatedly brought to ALI to study exposure to specified atmospheric conditions. During ALI conditions, cells cultured on porous (membrane) inserts are deprived of medium in the apical compartment, exposing them to the atmosphere and requiring them to take up nutrients from the basolateral compartment. These conditions allow the cell layer to mimic in vivo functionality more closely than immortal cell lines in submerged culture [97]. Despite the obvious advantages offered by this model, it should be noted that 2D monolayers differ significantly from in vivo epithelial structures and differentiated ALI cultures. In a study by Clark et al. [98], the transcriptional profiles of a 2D monolayer and a differentiated ALI structure, both consisting of primary human tracheal epithelial cells, were compared in an unstimulated state and following stimulation with flagellin. Flagellin is the main component of flagella and is primarily recognised by and activates downstream signalling of Toll-like receptor 5. The most important source of variation between the transcriptional profiles appeared to be the culture method (2D monolayer versus ALI culture), as opposed to flagellin stimulation. 2D monolayers displayed a more extensive immune response upon flagellin exposure compared to ALI cultures. The monolayer-stimulated transcriptional profile was characterised by upregulation of a wide array of genes involved in wound repair, inflammation and immunity, whereas a more modest response was observed in ALI cultures. This muted response might represent a more in vivo-like behaviour and serve as a protection against an excessive pro-inflammatory reaction. Research on the host response upon stimulation with bacterial material or allergens is not limited to epithelial structures but can be expanded into the immune compartment. Suspension cultures of immune cells can be used to examine immunological responses to CRS-associated allergens. Larsen et al. [99] used UV-killed commensal and pathogenic airway bacteria to examine dendritic cell maturation and pro- and anti-inflammatory immune stimulation to shed light on the role of changes in the airway microbiota in inflammatory airway diseases. Li et al. [100] used this method to study the reaction of peripheral blood monocytic cells to heat-killed *S. aureus* and observed downregulation of T_*H*_1 responses, offering a possible explanation for the T_*H*_2 inflammatory environment often observed in CRS.

#### Between 2D and 3D: differentiated cell layers

A well-established model for airway epithelium research is cell culture with differentiated airway epithelium at ALI. In practice, airway epithelial cells (cell lines or primary cells) are seeded on a microporous membrane and kept under submerged conditions, with medium in both apical and basal compartments, until full confluence (2D conventional monolayer). When the monolayer has reached confluency, the medium on the apical side is removed. This leaves the apical side exposed to air, while nutrients and fluids need to be obtained from the basal compartment. Apical exposure to air results in more representative mucus production compared to submersed culture in an airway epithelial cell line (Calu-3) [96]. At this point, the monolayer can differentiate in a period of 2–4 weeks from a 2D conventional monolayer to a polarised, pseudostratified epithelial structure composed of basal cells, ciliated cells and mucin-secreting goblet cells [62]. Differentiated cell layers are the follow-up stage of 2D monolayers but can be distinguished from the latter by their morphology and functionality. The multi-layered epithelial structure mimics the physiological barrier function in the respiratory tract with its functional tight junctions. ALI cultures have a more representative phenotype of the in vivo epithelium and can be maintained for a longer period (weeks to months) in comparison with medium-submerged monolayers [95, 101, 102]. A fully automated model for ALI culture of primary cells, called CULTEX^®^ LTC-C (long-term cultivation continuous) has been developed by Aufderheide et al. [103]. The CULTEX^®^ LTC-C provides temperature control, semi-continuous medium supply and automated medium-level adjustment, hereby increasing the quality and reproducibility of the culturing process, while reducing the labour intensity of conventional cell culture. Other non-automated differentiated ALI models of the upper respiratory tract are commercially available. The MucilAir™-HF model consists of primary nasal cells co-cultured with human fibroblasts, and the EpiAirway™ model contains primary human tracheal/bronchial cells and also exists as a co-culture with fibroblasts. Blume et al. [94] developed a dynamic microfluidic model of differentiated primary airway epithelial cells at ALI that allows close monitoring of immune responses of the cell layer. These different airway epithelial models allow research on exposure to environmental allergens [94, 104–106] and could be optimised for disease-specific purposes. Epithelial response to the presence of bacteria can be simulated by exposure to bacterial cell wall components such as lipopolysaccharide (LPS), flagellin or bacterial toxins such as *S. aureus* enterotoxin B and *S. aureus* alpha toxin, upon which innate immune responses of CRS-derived cultures can be compared to those originating from healthy controls [54, 69, 98].

#### Multicellular models and 3D scaffolds

The combination of different cell types within a model is a strategy to more accurately represent the host microenvironment and the interactions therein. Benam et al. [107] developed an ALI lung-on-chip model of human bronchioles with epithelial and endothelial tissue to investigate inflammatory reactions in chronic obstructive pulmonary disease and asthma. This model comprised an endothelial cell layer with primary cells to mimic the expression of receptors required for neutrophil rolling (transient adhesion to E-selectin) and more firm adhesion (to ICAM-1), as observed during in vivo inflammation. Recently, an immunocompetent 3D model at ALI of the upper respiratory tract was developed [87]. The tri-culture model consisted of three biomimetic electrospun scaffolds upon which, from bottom to top, respectively fibroblasts (MRC-5 cell line), dendritic cells (isolated from peripheral blood lymphocytes) and differentiated epithelial cells (Calu-3 cell line) were grown. As a proof of concept of immune response, dendritic cells were shown to migrate through the scaffolds and cell layers upon allergen exposure.

### Host-microbe co-culture models

Host-microbe co-culture models offer the possibility to investigate host-microbe interaction in a controlled environment with reduced complexity compared to human and animal models. This facilitates control of specific conditions and exploration of novel therapeutic strategies. The improved technical ability to create in vitro differentiated host models and the combination with multiple omics techniques allows improved insight in host-microbe crosstalk during infection and (inflammatory) responses [35, 68, 108]. Current co-culture models of the respiratory tract have mainly focused on colonisation with a single pathogenic [68, 90, 108, 109] or probiotic species [110]. The models can be used to monitor short-term host-microbe interactions, such as innate immune responses of the epithelial cells and physiological changes of bacterial and epithelial cells such as cytokine production and tight junction functionality [68, 90]. Starner et al. [109] developed the first in vitro model for bacterial biofilm formation on polarised human airway epithelia. Differentiated Calu-3 epithelial cells at ALI were used in co-culture with *H. influenzae* for four consecutive days, during which the epithelial cells remained viable. The model allowed follow-up of innate immune responses and biofilm formation. A similar set-up was used to study *S. aureus* colonisation of airway epithelial cells and the physiological changes that occurred during the colonisation process [90]. However, the epithelial layer was completely disrupted within 1 day after inoculation of wild-type *S. aureus*. Baddal et al. [108] have used simultaneous whole genome transcriptional profiling of host cells and a *H. influenzae* strain invading primary differentiated airway epithelium. Pathogen-mediated signalling pathways and significant dysregulation of the target cytoskeletal network upon intracellular infection were identified. This approach also enabled discovery of host adaptation pathways in the pathogen and metabolic signature traits of nasopharyngeal colonisation. Another co-culture model to study early innate immune responses of airway epithelium upon bacterial infection was developed by Kohanski and Lane [68]. The host environment was mimicked by an ALI-differentiated structure of sinonasal epithelial cells from CRS patients with and without nasal polyps and non-CRS controls. Distinguished inflammatory responses could be observed for the CRS subtypes upon infection with *S. aureus*, suggesting that the primary material has an inflammatory memory and could therefore be used to mimic the host environment more adequately. A submerged monolayer of human bronchial epithelium cells (HBE014 cell line) was used to investigate phenotype switching to small colony variants of *S. aureus* upon internalisation in epithelial cells and the subsequent effect on the immune response [111]. It was shown that intracellular *S. aureus* did not elicit excretion of the pro-inflammatory IL-6, contrarily to extracellular *S. aureus*. Cytotoxic effects were neither induced. A similar approach was followed to examine the differences between wild type and small colony producing *S. aureus* in epithelial cell invasion and subsequent host immune response [112]. Small colony variant producing *S. aureus* was shown to elicit a less widespread innate immune response at similar colonisation rates, thus evading clearance by the host. These findings could help explain bacterial persistence in CRS [111, 112].

Existing models are limited in the time frame of the host-microbe co-culture and the ability to adequately mimic the URT microenvironment; due to cell toxicity, the duration of host-live microbe co-cultures with direct contact between mammalian cells and bacteria is currently limited to hours or days [68, 90, 108]. This duration can be used for investigation of initial attachment and colonisation of bacteria but does not permit examination of bacterial persistence strategies. Starner et al. [109] inoculated *H. influenzae* with a multiplicity of infection of 20 bacteria/host cell and retained viable epithelial cells until 4 days post-inoculation. Remarkably, Ren et al. [113, 114] have used the EpiAirway™ model for co-culture of the host compartment with *H. influenzae*, extending the co-culture period to 10 days. Key aspects for success were the low multiplicity of infection (0.01–10 bacteria/host cell) and daily washes with phosphate-buffered saline. Another limitation to current models is failure of the host compartment to adequately mimic the host microenvironment as most often undifferentiated host cell monolayers or differentiated epithelial cell cultures are used, without taking into account tissue-specific mechanical or chemical stimuli. Organ-on-chip models offer a way to take these stimuli into account mimicking the spatial structures and mechanical stimuli observed in host tissue in microfluidic devices [107, 115, 116]. The ALI culture method, also applied in these devices, provides conditions for co-culture resembling the in vivo situation, where bacteria are exposed to atmospheric conditions and are obliged to acquire nutrients and growth factors through the epithelial cell layer.

### Microbial co-culture models

The majority of in vitro studies on URT infections have focused on competition assays between two microbial species. However, model systems for the URT microbiota in health and CRS are required to gain mechanistic insight in the factors that influence microbial colonisation behaviour, growth characteristics and community composition and the impact thereof on the expression of pathogenic traits and disease aetiology and evolution.

Mucostasis, a result of reduced ciliary movement and ostium obstruction, is commonly observed in the paranasal sinuses of CRS patients and can result in the accumulation of microorganisms and other immunogenic compounds in the paranasal sinuses. Furthermore, it alters the spatial structure of the growth surface of the bacterial community, a parameter known to impact colonisation behaviour [117]. The altered, inflammatory microenvironment can influence the community composition, as some species are more susceptible to host defence mechanisms than others. Microbial co-culture models can range from two-species co-cultivation over synthetic communities to natural communities. Simple co-cultivation models offer the advantage of a low complexity, high controllability and relative ease to analyse, but often lack representative in vivo behaviour. Natural communities typically display a higher level of complexity and have a broader and more representative functionality. Disadvantages are the lower controllability and reproducibility and difficulty of analysis. Synthetic communities combine the advantages of both systems by having a broader functionality than simple co-cultures, while being more controllable and reproducible than complex communities [118]. Recent research on healthy and CRS sinonasal microbiota indicates the existence of community state types [119] or disease subgroups [9] with distinctive bacterial compositions. This offers the opportunity to develop different synthetic communities associated with CRS phenotypes and opens the way to precision research and medicine.

Competition assays between two microbial species enable the investigation of specific interaction between these species in a relatively simple set-up and may lead to discovery of novel strategies to prevent pathogenic infections. Biofilm co-culture experiments are especially relevant in CRS research and have for example been used to study species interactions before and during the biofilm state. In vitro studies demonstrated that *Staphylococcus epidermidis* can have an inhibitory effect on *S. aureus* biofilm formation [120, 121], partly explaining the negative correlation between *S. epidermidis* colonisation and *S. aureus* carriage observed in hospitalised patients [122]. *S. aureus* and *H. influenzae* are regularly observed together in CRS-associated biofilms [85]. The interactions between *S. pneumoniae* and *H. influenzae* were investigated during pre-biofilm planktonic growth and biofilm formation, and the inclination towards antagonism or mutualism was shown to be largely dependent on environmental parameters such as growth phase and nutrient availability [123]. Interspecies interactions in *S. aureus* biofilm formation and co-aggregation were examined between nostril-dwelling *S. aureus* and *Propionibacterium* spp., where a porphyrin compound excreted by *Propionibacterium* spp. was found to induce *S. aureus* aggregation and biofilm formation [124]. More simplistic co-culture models include agar competition experiments and broth co-culture. Cope et al. [89] demonstrated interspecies interactions and their influence on virulence gene expression between *H. influenzae* and *S. pneumoniae* both in vitro and ex vivo in human CRS sinus tissue. *H. influenzae* type IV pili, important for epithelial colonisation and biofilm formation, were only expressed in co-culture in vitro, whereas *S. pneumoniae* virulence factors associated with acute infection and epithelial damage were downregulated, adhering to a more chronic infection model. Yan et al. [25] first studied bacterial community differences between persistent and non-persistent *S. aureus* carriers and found a co-occurrence between *S. aureus* and *Corynebacterium accolens*, while *Corynebacterium pseudodiphteriticum* was observed more often in non-persistent carriers. Growth interaction assays on agar plates revealed that *S. aureus* and *C. accolens* supported each others’ growth, while *C. pseudodiphteriticum* growth was less supported by *S. aureus* and *C. pseudodiphteriticum* even inhibited *S. aureus* [25].

CRS is a multifactorial disease, as elaborated previously, and despite the established importance of *S. aureus* and other pathogenic species in the disease, it is unlikely to be caused by a single infectious microorganism. It is rather to be expected that, on the bacterial side, an imbalance in the microbial community plays a role. The reductionist approach of two or multiple species co-cultures is unable to answer research questions on colonisation and growth dynamics in the complex, pre-colonised mucosal environment seen in CRS. Considering CRS as a poly-microbial disease, research on complex communities, deciphering co-occurrence patterns, mutualistic interactions and the importance of the relative abundance of the community members, is indispensable to understand CRS and find appropriate treatment or preventive strategies [125]. Reduced community evenness has been shown to decrease resistance to invasion [126], which could facilitate pathogen colonisation in CRS community in state of dysbiosis and further CRS pathogenesis. It is in this ecosystemic framework that further CRS research initiatives should take place.

Bacterial co- or mixed culture experiments have the major disadvantage of omitting the host’s immune responses and thus omitting within-host competition behaviour [127] and more broadly the effects of microbial co-infection [128, 129]. Within-host competition is a strategy during which a species elicits an immune response to which it is resistant itself, but that is able to clear the niche of competing organisms. These off-target immune defences comprise cross-reacting antibodies, recruitment of polymorphonuclear cells and production of antimicrobial proteins. Immunological effects can severely influence and even revert competition outcomes compared to in vitro observations [127].

## Conclusion

Chronic rhinosinusitis is a multifactorial disease of uncertain aetiology, driven by host immune responses, microbial dysbiosis and exposure to environmental irritants. Mechanistic research to unravel aetiopathologic pathways of this disease and find innovative prophylactic or treatment approaches requires in vitro models of the affected tissues and their resident microbiota. Current models are either limited to host cells, competition assays between few airway species or simple host-pathogen co-cultures. To adequately mimic host and microbial behaviour in CRS, a less reductionist approach is needed, in particular on the microbial aspect. A polymicrobial, dynamic community is a prerequisite to investigate pathogen colonisation and inflammatory responses elicited in the host. Long-term models are needed to analyse shifts in the microbial community composition and functionality during CRS and how these communities can be modulated to improve chronic inflammation and restore host-microbe balance in the sinonasal cavities.

## Abbreviations

ALI: Air-liquid interface
AMP: Antimicrobial protein
ASL: Airway surface liquid
CRS: Chronic rhinosinusitis
CRSsNP: Chronic rhinosinusitis without nasal polyps
CRSwNP: Chronic rhinosinusitis with nasal polyps
IFN: Interferon
Ig: Immunoglobulin
IL: Interleukin
LPS: Lipopolysaccharide
NP: Nasal polyps
PLUNC: Palate, lung, nasal epithelial clone
SPLUNC-1: Short palate, lung, nasal epithelial clone 1
T2R: Type 2 receptor
URT: Upper respiratory tract
